# COVID-19 in India: Statewise Analysis and Prediction

**DOI:** 10.2196/20341

**Published:** 2020-08-12

**Authors:** Palash Ghosh, Rik Ghosh, Bibhas Chakraborty

**Affiliations:** 1 Department of Mathematics Indian Institute of Technology Guwahati India; 2 Centre for Quantitative Medicine Duke-National University of Singapore Medical School Singapore Singapore; 3 Centre for Quantitative Medicine & Programme in Health Services and Systems Research Duke-National University of Singapore Medical School Singapore Singapore; 4 Department of Statistics and Applied Probability National University of Singapore Singapore Singapore; 5 Department of Biostatistics and Bioinformatics Duke University Durham, NC United States

**Keywords:** COVID-19, disease modeling, 30-day prediction, logistic model, exponential model, SIS model, daily infection rate

## Abstract

**Background:**

The highly infectious coronavirus disease (COVID-19) was first detected in Wuhan, China in December 2019 and subsequently spread to 212 countries and territories around the world, infecting millions of people. In India, a large country of about 1.3 billion people, the disease was first detected on January 30, 2020, in a student returning from Wuhan. The total number of confirmed infections in India as of May 3, 2020, is more than 37,000 and is currently growing fast.

**Objective:**

Most of the prior research and media coverage focused on the number of infections in the entire country. However, given the size and diversity of India, it is important to look at the spread of the disease in each state separately, wherein the situations are quite different. In this paper, we aim to analyze data on the number of infected people in each Indian state (restricted to only those states with enough data for prediction) and predict the number of infections for that state in the next 30 days. We hope that such statewise predictions would help the state governments better channelize their limited health care resources.

**Methods:**

Since predictions from any one model can potentially be misleading, we considered three growth models, namely, the logistic, the exponential, and the susceptible-infectious-susceptible models, and finally developed a data-driven ensemble of predictions from the logistic and the exponential models using functions of the model-free maximum daily infection rate (DIR) over the last 2 weeks (a measure of recent trend) as weights. The DIR is used to measure the success of the nationwide lockdown. We jointly interpreted the results from all models along with the recent DIR values for each state and categorized the states as severe, moderate, or controlled.

**Results:**

We found that 7 states, namely, Maharashtra, Delhi, Gujarat, Madhya Pradesh, Andhra Pradesh, Uttar Pradesh, and West Bengal are in the severe category. Among the remaining states, Tamil Nadu, Rajasthan, Punjab, and Bihar are in the moderate category, whereas Kerala, Haryana, Jammu and Kashmir, Karnataka, and Telangana are in the controlled category. We also tabulated actual predicted numbers from various models for each state. All the *R*^2^ values corresponding to the logistic and the exponential models are above 0.90, indicating a reasonable goodness of fit. We also provide a web application to see the forecast based on recent data that is updated regularly.

**Conclusions:**

States with nondecreasing DIR values need to immediately ramp up the preventive measures to combat the COVID-19 pandemic. On the other hand, the states with decreasing DIR can maintain the same status to see the DIR slowly become zero or negative for a consecutive 14 days to be able to declare the end of the pandemic.

## Introduction

### Background

The world is now facing an unprecedented crisis due to the novel coronavirus, first detected in Wuhan, China in December 2019 [1]. The World Health Organization (WHO) defined coronavirus as a family of viruses that range from the common cold to the Middle East respiratory syndrome coronavirus and the severe acute respiratory syndrome coronavirus [2]. Coronaviruses circulate in some wild animals and have the capability to transmit from animals to humans. These viruses can cause respiratory symptoms in humans, along with other symptoms of the common cold and fever [3]. There are no specific treatments for coronaviruses to date. However, one can avoid infection by maintaining basic personal hygiene and social distancing from infected persons.

**Figure 1:**
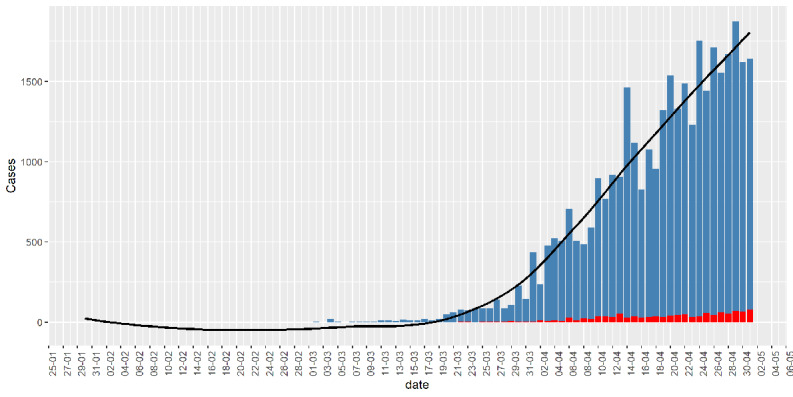
Bar chart of daily infected cases (blue) in India. Red bar denotes death. The black curve is a fitted smooth curve on the daily cases.

The WHO declared the coronavirus disease (COVID-19) as a global pandemic on March 11, 2020 [4]. The disease has spread across 212 countries and territories around the world, with a total of more than 3 million confirmed cases [5,6]. In India, the disease was first detected on January 30, 2020, in Kerala in a student who returned from Wuhan [7,8]. The total (cumulative) number of confirmed infected people is more than 37,000 to date (May 3, 2020) across India. The bar chart in Figure 1 shows the daily growth of the COVID-19 cases in India. After the first 3 cases from January 30 to February 3, 2020, there were no confirmed COVID-19 cases for about a month. The COVID-19 cases appeared again from March 2, 2020, onwards. These cases are related to people who have been evacuated or have arrived from COVID-19–affected countries. From March 20, 2020, onwards, there is an exponential growth in the daily number of COVID-19 cases at the pan-India level.

There are four stages of COVID-19 depending on the types of virus transmission [9,10]. During the first stage, a country or region experiences imported infected cases with travel history from virus-hit countries. During the second stage, a country or region gets new infections from persons who did not have a travel history but came in contact with persons defined in stage 1. Stage 3 is community transmission; in this period, new infection occurs in a person who has not been in contact with an infected person or anyone with a travel history of virus-hit countries. At stage 4, the virus spread is practically uncontrollable, and the country can have many major clusters of infection.

Many news agencies are repeatedly saying or questioning whether India is now at stage 3 [9,11,12]. In reality, different Indian states are or will be at various stages of infection at different points in time. Labeling a COVID-19 stage at the pan-India level is problematic. It will spread misinformation to common people. Those states that are at stage 3 require more rapid action compared to others. On the other hand, states that are in stages 1 and 2 need to focus on stopping the community spread of COVID-19.

In this paper, we first discuss the importance of statewise consideration, contemplating all the states together. Second, we will focus on the infected people in each state (considering only those states with enough data for prediction) and build growth models to predict infected people for that state in the next 30 days.

### Why Statewise Consideration?

India is a vast country with a geographic area of 3,287,240 square kilometers and a total population of about 1.3 billion [13]. Most of the Indian states are quite large in geographic area and population. Analyzing coronavirus infection data, considering the entirety of India to be on the same page may not provide us the right picture. This is because the first infection, new infection rate, progression over time, and preventive measures taken by state governments and the common public for each state are different. We need to address each state separately. It will enable the government to use the limited available resources optimally. For example, currently, Maharashtra already has more than 10,000 confirmed infected cases, whereas West Bengal has less than 800 confirmed cases (May 1, 2020). The approaches to addressing the two states must be different due to limited resources. One way to separate the statewise trajectories is to look at when each state was first infected.

**Figure 2:**
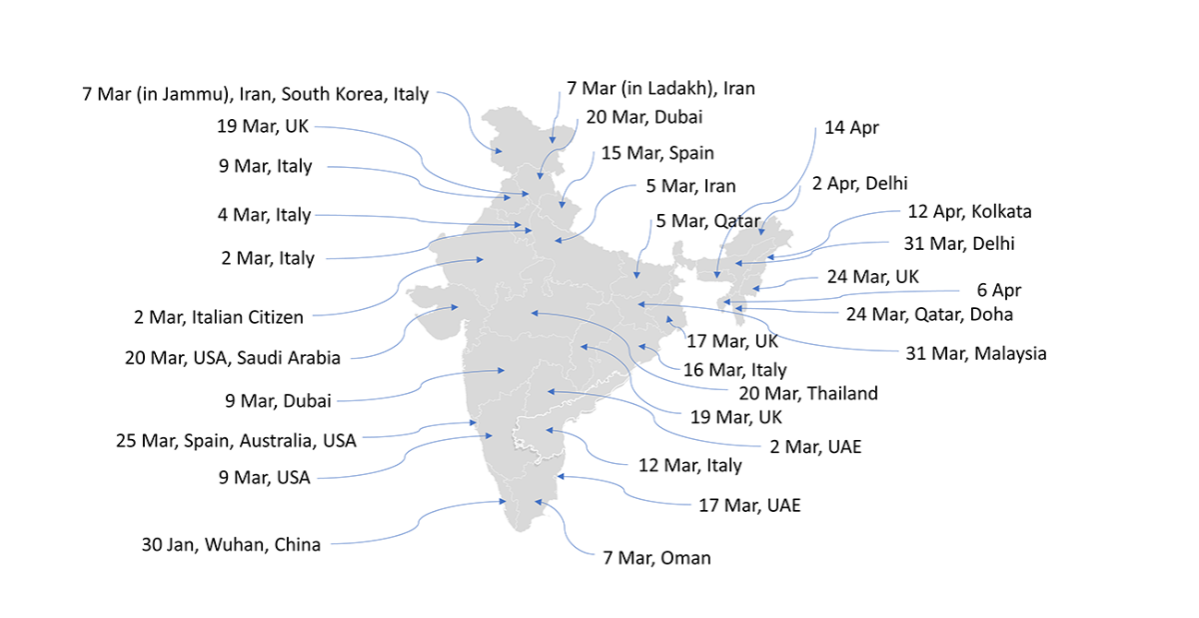
When the first case in each state happened with their travel histories. UAE: United Arab Emirates.

In Figure 2, we present the first infection date along with the infected person’s travel history in each of the Indian states. All the states and the union territories, except Assam, Tripura, Nagaland, Meghalaya, and Arunachal Pradesh, observed their first confirmed infected case from a person who had travel history from one or more already COVID-19–infected countries. The Indian government imposed a complete ban on international flights to India on March 22, 2020 [14]. Figure 2 justifies government action to international flight suspension. Had it been taken earlier, we could have restricted the disease to only a few states compared to the current scenario.

**Figure 3:**
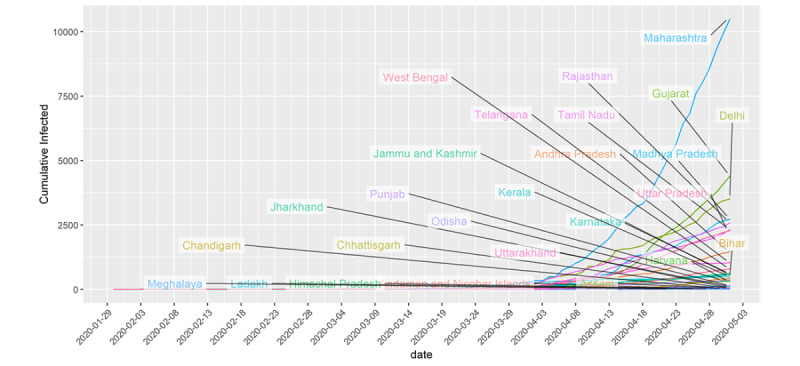
Cumulative number of infected people over time in states with at least 10 infected cases.

Figure 3 shows the curve of the cumulative number of infected people in those Indian states having at least 10 total infected people. Currently, Maharashtra, Delhi, Gujarat, Tamil Nadu, Madhya Pradesh, Rajasthan, and Uttar Pradesh are the states where the cumulative number of infected people have crossed the 2000 mark, with Maharashtra having more than 10,000 cases. Kerala, the first state to have a COVID-19 confirmed case, seems to have restricted the growth rate. There are few states with cumulative infected people in the range of 500-1500. Depending on how those states strictly follow the preventive measures, we may see a rise in the confirmed cases.

### Preventive Measures

In Textbox 1, we list the major preventive measures taken by the Indian Government [15].

**List of major preventive measures taken by the Indian Government.**

**January 25-March 13, 2020**

Health screenings at airports and border crossings

**February 26-March 20, 2020**

Introduction of quarantine policies: gradually for passengers coming from different countries

**February 26-March 13, 2020**

Visa restrictions: gradually for different countries

**March 5, 2020**

Limit public gatherings (closure of some selected public institutions like museums, religious places, and postponing of several local elections to stop public gatherings)

**March 11, 2020**

Border checks

**March 13-15, 2020**

Border closure

**March 16, 2020**

Limit public gatherings (ban on all sorts of public gatherings and meetings, and stopping people from making any congregation)

**March 18, 2020**

Travel restrictions

**March 20, 2020**

Testing for the coronavirus disease (before this point, only people who had traveled from abroad were tested; this point onwards, testing was also introduced for symptomatic contacts of laboratory-confirmed cases, symptomatic health care workers, and all hospitalized patients with severe acute respiratory illness)

**March 22, 2020**

Flight suspensions

**March 22, 2020**

Cancellation of passenger train services until March 31, 2020

**March 24, 2020**

Suspension of domestic airplane operations

**March 25, 2020**

21-day lockdown of entire country

**March 25, 2020**

Cancellation of passenger train services extended to April 14, 2020

**March 30, 2020**

Increase of quarantine/isolation facilities

**April 14, 2020**

Extension of lockdown until May 3, 2020

**May 1, 2020**

Extension of lockdown until May 17, 2020

## Methods

### Data Source

We have used Indian COVID-19 data available publicly. The three primary sources of the data are the Ministry of Health and Family Welfare, India [16]; COVID-19 India [17]; and Wikipedia [18].

### Statistical Models

In this paper, we consider the exponential model, the logistic model, and the susceptible-infectious-susceptible (SIS) model for COVID-19 pandemic prediction at the state level. These models have already been used to predict epidemics like COVID-19 around the world, including in China, and for the Ebola outbreak in Bomi, Liberia in 2014 [19-21]. See Multimedia Appendix 1 [20-22] for details about the models.

#### Using the Models in State-Level Data

The previously mentioned three models will provide a different prediction perspective for each state. The exponential model–based prediction will give a picture of what could be the cumulative number of infected people in the next 30 days if we do not take any preventive measures. We can consider the forecast from the exponential model as an estimate of the upper bound of the total number of infected people in the next 30 days. The logistic model–based prediction will capture the effect of preventive measures that have already been taken by the respective state governments as well as the central government. The logistic model assumes that the infection rate will slow down in the future with an overall “S” type growth curve. In other words, the logistic model tries to explore a situation where there is a full lockdown in the country, leading to an extreme restraint on the people’s movement, hence reducing the rate of infection considerably. Under the effective implementation of the lockdown, it is appropriate to use a logistic model. In this scenario, many people have already been infected; the virus may find it hard to spot more susceptible people. Thus, the virus slows down its spread, causing the flattening in the S-curve at a later stage. Several research papers have used the logistic model in the context of COVID-19 [23-26].

The purpose of the SIS model is to reflect the effect of the major preventive measure like the nationwide 21-day lockdown from March 25 to April 14, 2020. The lockdown was extended in two phases: (1) until May 3 and (2) then until May 17, 2020, with some relaxation [27,28]. The SIS model is critically dependent on the infection-rate parameter (β). It is defined as the number of people infected per unit time from an infected person. Note that this parameter is subject to change due to the effect of lockdown and other preventive measures to ensure social distancing. When people are at home, the infection rate is expected to be on the lower side. The other parameter in the SIS model is 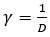 with *D* being the recovery time. We have considered 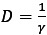 = 14 days [29,30]. In this study, to make the SIS model simple, we assumed that the number of births and deaths in a state are the same.

### Study the Effect of Lockdown Using the Daily Infection Rate and SIS Model

Kumar et al [31] reported the estimated number of people that a person may *come in contact with within* a day (24 hours) in a rural community in Haryana, India to be 17. They defined *contact* as having a face-to-face conversation within 3 feet, which may or may not have included physical contact. The estimate of the contact-rate parameter from their paper is 0.70. In practice, only some of all the people who come in *contact* with a person infected with COVID-19 may be actually infected by the virus. Note that India has already taken many preventive measures to ensure social distancing. In the current scenario, the infection rate based on Kumar et al’s [31] study could be an overestimate of its present value. However, despite nationwide lockdown, banks, hospitals, and grocery stores are still open to cater to the essential needs of people. We consider here two approaches to study the effect of lockdown and other preventive measures jointly in each state. *First*, we plot the daily infection rates (DIRs) for each state. The DIR for a given day is defined as:

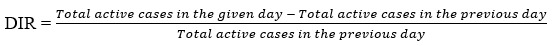



The DIR takes a positive value when we see an increase in active COVID-19 cases from yesterday, the zero value in case of no change in the number of active cases from yesterday, and a negative value when the total number of active cases decreases from the previous day. A DIR value can be more than 1 also, particularly during initial days of infection in a state. For example, when the total number of active cases increases from 5 yesterday to 20 today, then the DIR value is (20 – 5) / 5 = 3. The visual trends in infection rates can explain whether the COVID-19 situation is under control or not in a specific state. A state where DIRs are declining for the last few days indicates that the situation is improving. However, a certain jump in infection rates could inform us that there could be cases of COVID-19 that are underreported. We need to search for infected clusters as quickly as possible. *Second*, we have incorporated a fitted SIS curve (fitted via a nonlinear least squares approach), a close representation of the observed number of cases (red curve) for each state. The estimated values of the basic reproduction number (R_0_) from the SIS model are also reported for each state. Here, 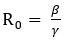 from the SIS model. Using the SIS model, we have also considered four predicted curves of active infected patients with different infection rates. The four different infection rates used in the SIS model for prediction are the 25th, 50th, 75th, and 80th percentiles of the observed DIRs. We also plotted the observed active infected patients over time. A declining curve of observed active infected patients (red curve) can ensure that measures like lockdown and social distancing are working when all the infected cases are reported and tested. The different predicted lines, using the SIS model, may serve as reference frames to indicate whether the government needs to enforce the social distancing more stringently. For example, if the current part of the graph of observed active infected patients (red curve) is above the 75th percentile line, then there is a major concern for that state. We may need to increase the lockdown period in a state if we do not see a declining trend of observed active infected patients (red curve).

India implemented a nationwide lockdown on March 25, 2020. We first considered the incubation period of the novel coronavirus to study the effect of the lockdown. The incubation period of an infectious disease is defined as the time between infection and the first appearance of signs and symptoms [32]. Using the incubation period, health researchers can decide on the quarantine periods and halt a potential pandemic without the aid of a vaccine or treatment [33]. The estimated median incubation period for COVID-19 is 5.1 (95% CI 4.5-5.8) days, and 97.5% of those who develop symptoms will do so within 11.5 (95% CI 8.2-15.6) days of infection [34]. The WHO recommends that a person with laboratory-confirmed COVID-19 be quarantined for 14 days from the last time they were exposed to the patient [35]. Therefore, if a person was infected before the lockdown (March 25, 2020), they should not infect others except their family members if that person is entirely inside their house for more than 14 days. The WHO also recommends common people to maintain a distance of at least 1 meter from each other in a public place to avoid COVID-19 infection. The effective implementation of social distancing can stop the spread of the virus from an infected person, even when they are outside for some essential business. However, given a highly dense population in most of India, particularly in cities, it may not always be possible to maintain adequate social distance.

## Results

### Statewise Analysis and Prediction Report

In this section, we depend on inputs from the exponential, logistic, and SIS models along with DIRs for each state. Remembering the words of the famous statistician George Box “All models are wrong, but some are useful,” we interpreted the results from different models jointly. We consider different states with at least 300 cumulative infected cases. For each state, we present four graphs. We have used the state-level data until May 1, 2020. The first and second graphs are based on the logistic and the exponential models, respectively, with the next 30-day predictions. The third graph is the plot of DIRs for a state. Finally, the fourth graph is showing the growth of the active infected patients using SIS model prediction (*“pred”*) along with the observed active infected patients. Table 1 represents the 30-day prediction of the cumulative infected number of people for each state using the logistic model, the exponential model, and a data-driven combination of the two. The corresponding measures of goodness of fit (*R*^2^ and deviance) are presented in the table in Multimedia Appendix 1.

**Table 1:** Data-driven assessment and 30-day prediction using the logistic and exponential models, and their linear combination.

State	Observed cumulative cases (May 1, 2020)	Maximum DIR^a^ in the last 2 weeks	Estimated R_0_^b^ from SIS^c^ model (data until May 1, 2020)	Data driven assessment of COVID-19^d^ situation	30-day prediction (May 31, 2020)			Observed cumulative cases (May 31, 2020)	Assessment of observed cumulative cases with respect to (LC_pred_^e^, exponential)
					Logistic	Linear combination of logistic and exponential (LC_pred_)	Exponential (applicable only if the situation is severe)		
Andhra Pradesh	1463	0.17	3.22	Severe	2313	4725	16,502	3571	Below
Bihar	426	0.39	3.08	Moderate	16,452	16,472	16,502	3807	Below
Delhi	3515	0.17	2.94	Severe	4262	9650	35,957	19,844	Between
Gujarat	4395	0.27	3.50	Severe	5206	33,736	110,874	16,794	Below
Haryana	313	0.18	1.82	Controlled	321	590	1815	2091	Above
Jammu and Kashmir	614	0.09	2.66	Controlled	724	1124	5170	2446	Between
Karnataka	576	0.06	2.38	Controlled	3711	3711	3713	3221	Below
Kerala	497	0.18	1.96	Controlled	455	740	2040	1270	Between
Madhya Pradesh	2719	0.10	3.36	Severe	3030	6521	37,935	8089	Between
Maharashtra	10,498	0.15	3.50	Severe	17,115	43,963	196,103	67,655	Between
Punjab	357	0.14	2.52	Moderate	419	713	2517	2263	Between
Rajasthan	2584	0.12	2.94	Moderate	2821	6125	30,356	8831	Between
Tamil Nadu	2323	0.12	3.22	Moderate	2241	3967	16,624	22,333	Above
Telangana	1039	0.09	2.66	Controlled	1063	1631	7373	2698	Between
Uttar Pradesh	2281	0.13	2.52	Severe	3016	6566	30,326	8075	Between
West Bengal	795	0.17	3.22	Severe	1261	3225	12,815	5501	Between

^a^DIR: daily infection rate.

^b^R_0_: basic reproduction number.

^c^SIS: susceptible-infectious-susceptible.

^d^COVID-19: coronavirus disease.

^e^LC_pred_: linear combination prediction.

#### Maharashtra

**Figure 4:**
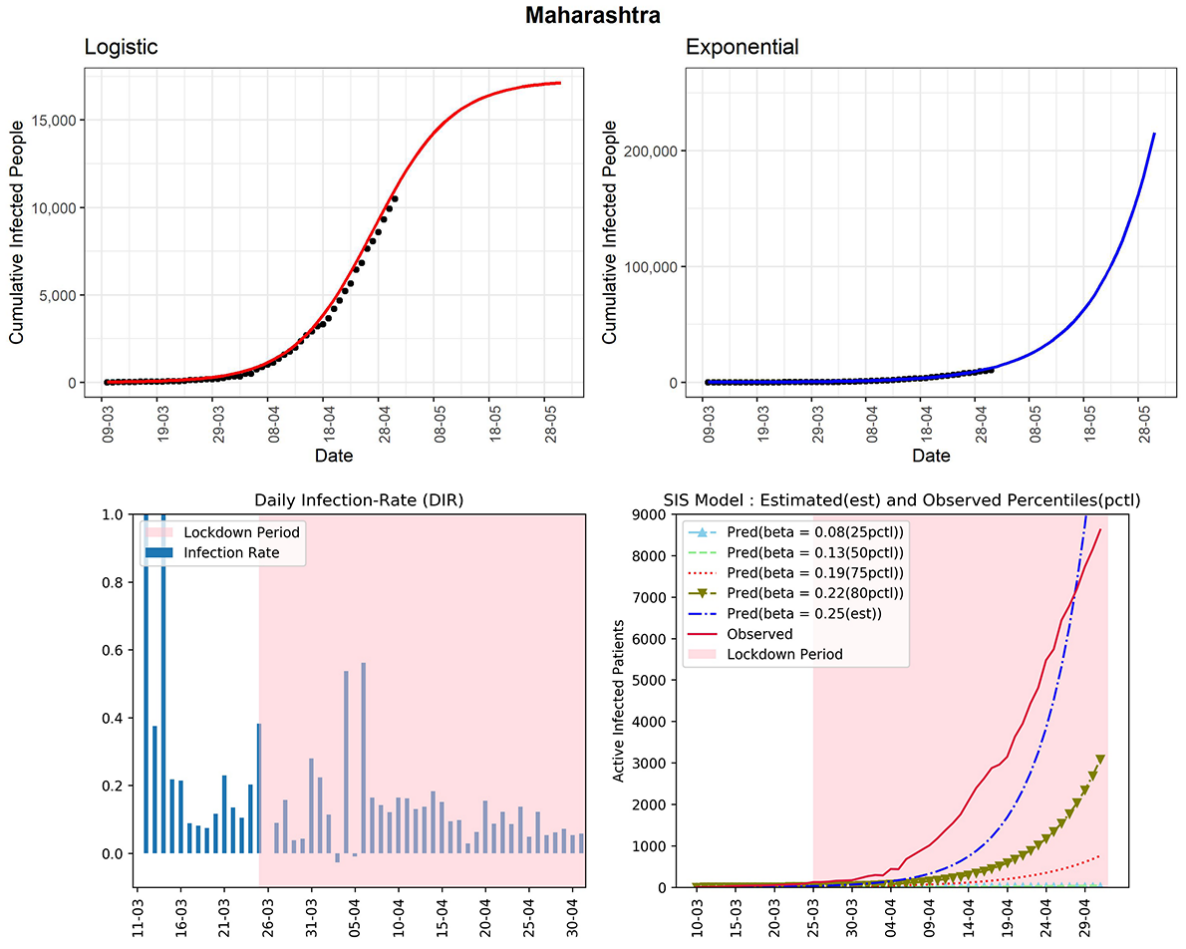
Graphs for the state of Maharashtra. SIS: susceptible-infectious-susceptible.

The situation in Maharashtra is currently very severe with respect to the active number of cases (see Figure 4). As of May 1, 2020, the total number of infected cases is 10,498. The logistic model indicates that, in another 30 days from now, the state could observe around 17,100 cumulative infected cases. The DIRs for this state were between 0.03 and 0.15 in the last 2 weeks, and it was more than 0.4 for 2 days at the beginning of April. Note that, for Maharashtra, the lower DIR values of 0.03 may not indicate a good sign since the total number of *active infected cases* is above 8000. Thus, a DIR value of 0.03 for a day implies 8000 x 0.03 = 240 new infected cases. The curves from the SIS model are alarming as the observed active infected patients (red line, fourth panel) line is far above the predicted line with estimated infection rate at the 80th percentile of observed DIRs (β=0.22). It is apparent from the graphs that even after 30 days of lockdown, Maharashtra has not seen any decline in the number of active cases. The estimated R_0_ for Maharashtra obtained from the fitted SIS model is 3.5, which is the highest among all the states. This may also indicate that there could be a large number of people who are in the community without knowing that they are carrying the virus. The state can be considered to be in stage 3.

#### Delhi

**Figure 5:**
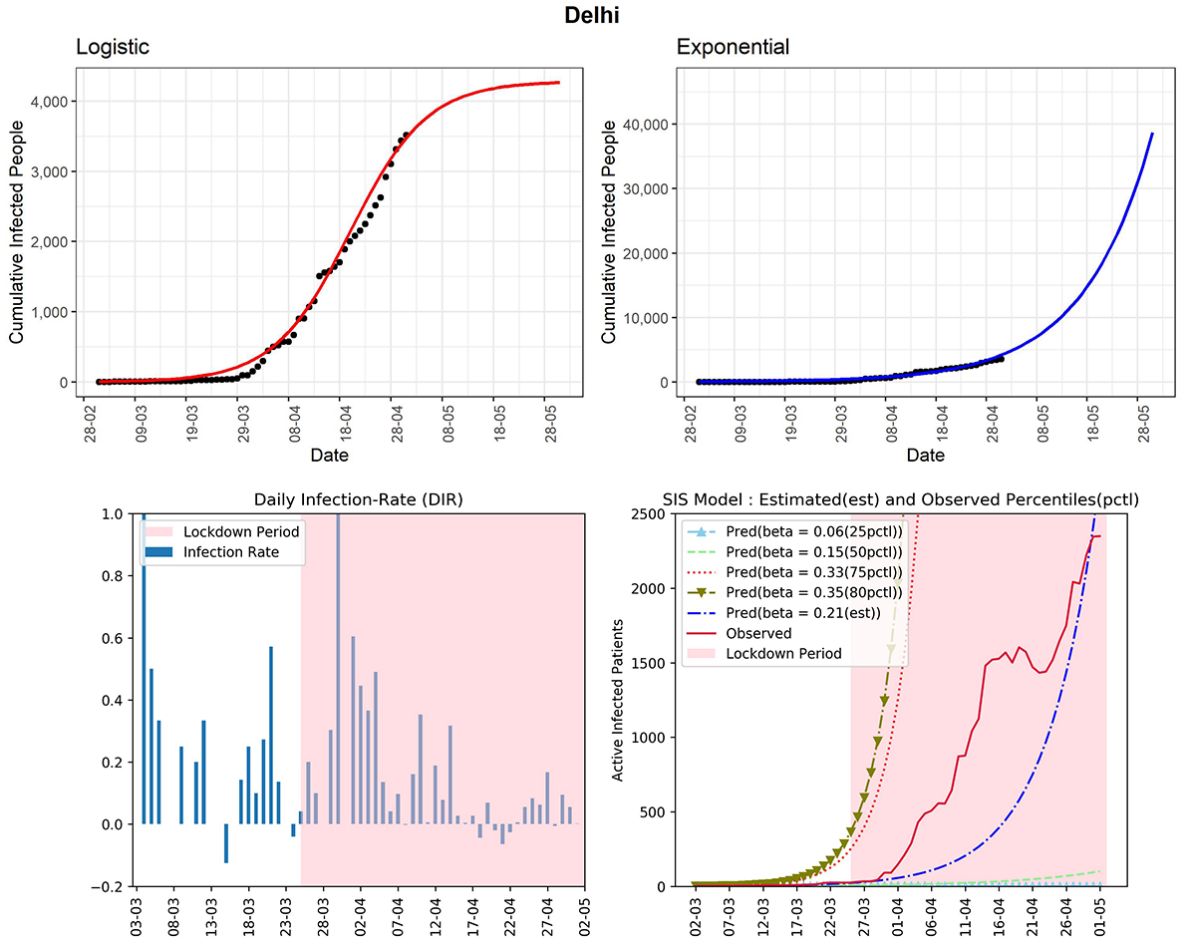
Graphs for the state of Delhi. SIS: susceptible-infectious-susceptible.

Delhi, being a state of high population density, has already observed 3515 confirmed COVID-19 cases (see Figure 5). Based on the logistic model, the predicted number of cumulative infected cases could reach around 4200 in the next 30 days. The DIR has not seen a downward trend in the past few days. The curve (red line, fourth panel) of observed active infected patients was showing a downward trend from April 20 to April 23, 2020. However, the same graph has picked up exponential growth in the last few days. This is an important observation that illustrates why we need a continuous downward trend of active cases for at least 14 days and that a slight relaxation may put a state in the same severe condition where it was earlier. The estimated R_0_ for the state obtained from the fitted SIS model being 2.94 is quite alarming. The observed DIR has been currently fluctuating between –0.06 and 0.17 in the last 2 weeks. The occasional high DIR may suggest that there could be many people who are in the community without knowing that they are already infected with COVID-19. The state could be heading to community spread of COVID-19 (stage 3).

#### Tamil Nadu

**Figure 6:**
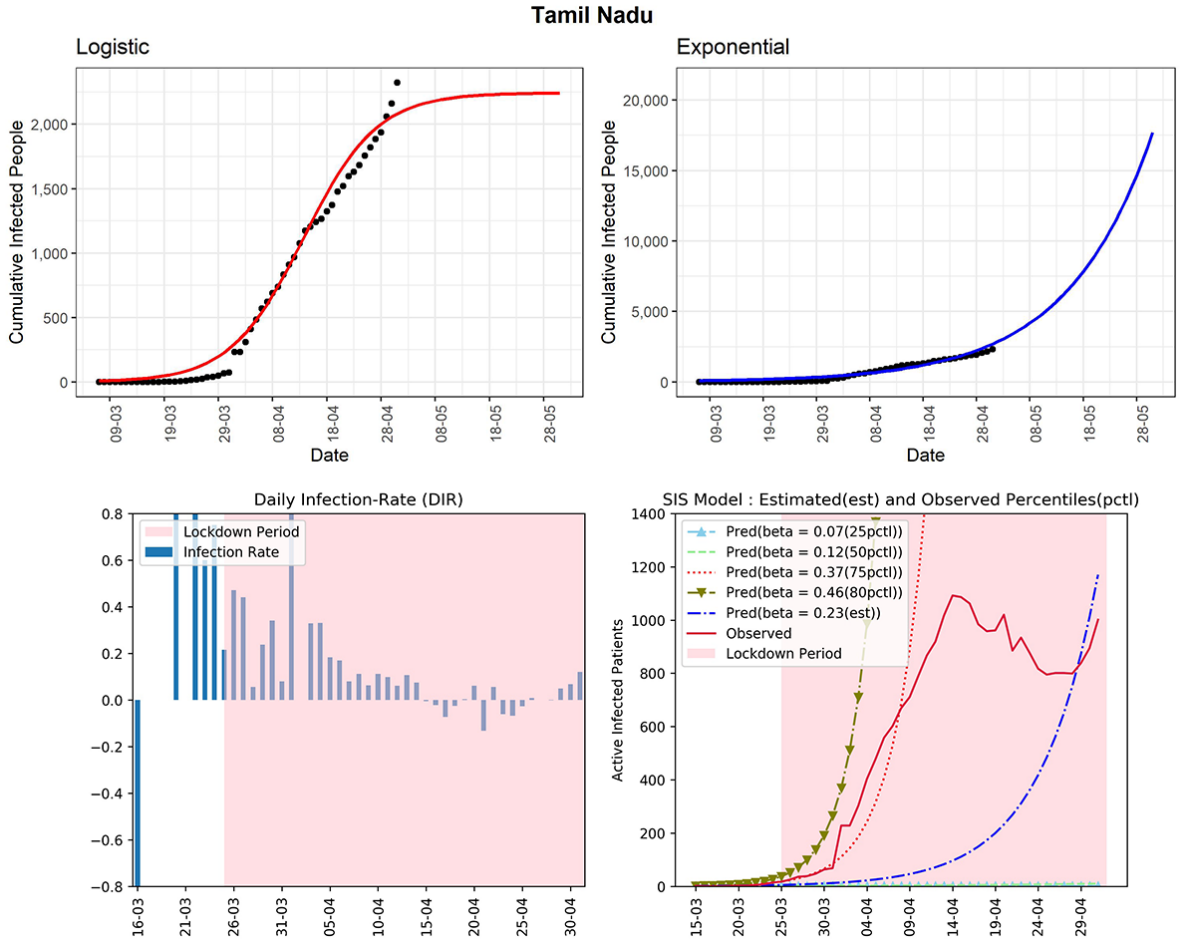
Graphs for the state of Tamil Nadu. SIS: susceptible-infectious-susceptible.

The cumulative infected cases in Tamil Nadu is 2323 (see Figure 6). The state has observed a high DIR of more than 0.7 for some days in March. Tamil Nadu is one of the states where the effect of lockdown is visible from the declining DIRs from the beginning to the end of April. However, there was again an increasing trend in DIR over the last 3 days. The DIRs were between –0.13 and 0.12 over the previous 2 weeks. The latter part of the curve (red line, fourth panel) of observed active infected patients is showing a decreasing trend first but then an increasing trend again. The estimated R_0_ for this southern state obtained from the fitted SIS model is 3.22, which is quite high. The preventive measures need to be maintained to bring down the active cases as well as to stop new infections in this state.

#### Madhya Pradesh

**Figure 7:**
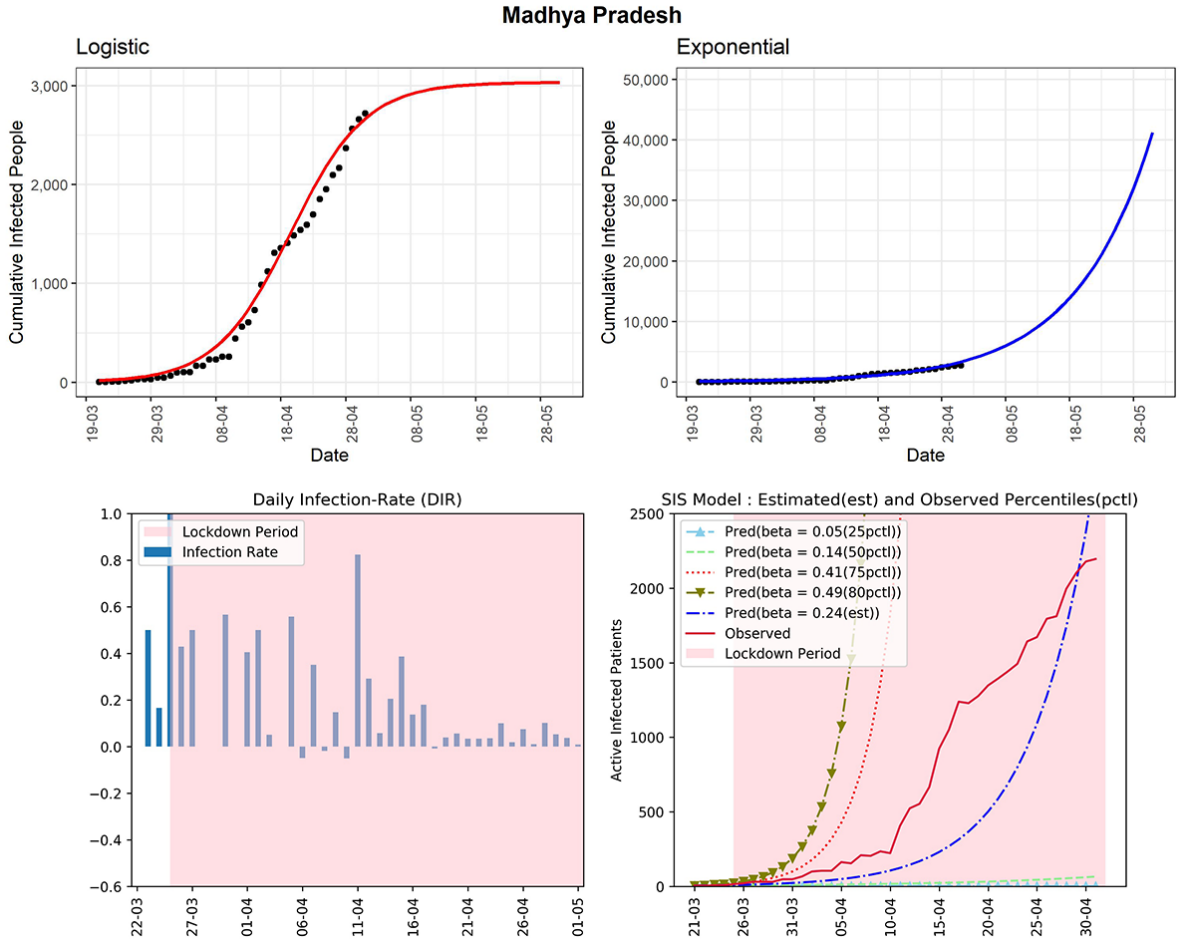
Graphs for the state of Madhya Pradesh. SIS: susceptible-infectious-susceptible.

This state currently has 2719 cumulative COVID-19 cases (see Figure 7). In the later part of the lockdown, after April 10, 2020, the state observed a few days with a DIR more than 0.4. Until now, there is no sight of a declining trend in the DIRs. The same type of conclusion can be drawn from the curves of the SIS model. The curve (red line, fourth panel) of observed active infected patients is in between the curves of the SIS model corresponding to the 50th-75th percentiles’ curves. The same curve is maintaining an exponential growth after April 10. Note that, for Madhya Pradesh, the 50th percentile of observed DIRs was 0.14, which is higher than the 50th percentile of some other states. The estimated R_0_ for this state obtained from the fitted SIS model was 3.36, which is pretty high. The high growth of active cases in the latter part of the lockdown is a major concern for this state. It could be a signal of a community spread of COVID-19.

#### Rajasthan

**Figure 8:**
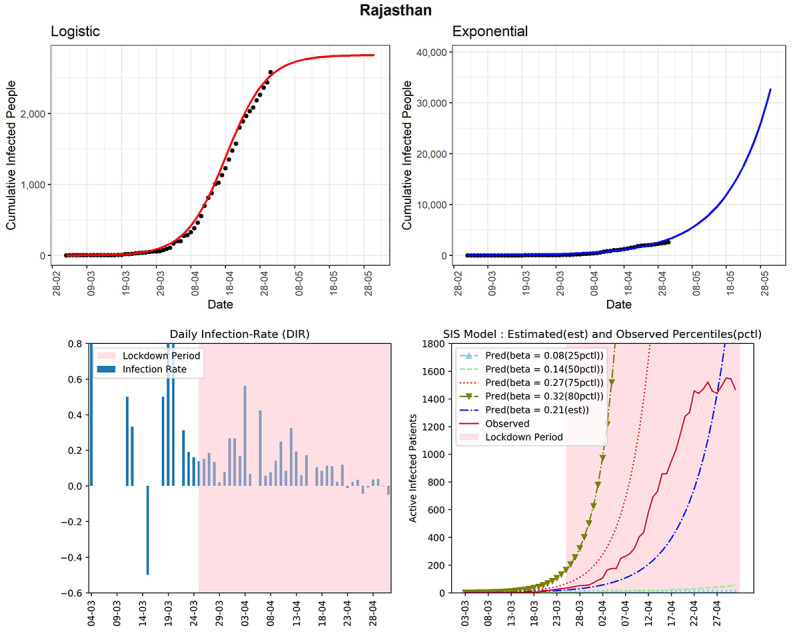
Graphs for the state of Rajasthan. SIS: susceptible-infectious-susceptible.

The western state of India, Rajasthan, reported 2584 cumulative infected COVID-19 cases (see Figure 8). The logistic model indicates that in another 30 days from now, the state could observe around 2800 cumulative infected cases. The state has seen a declining trend in the DIRs during the last part of April. The curve (red line, fourth panel) of observed active infected patients is increasing and is in between the curves of the SIS model corresponding to the 50th-75th percentiles of observed DIRs (0.14-0.27) using the SIS model. In the last 2 weeks, the DIRs for Rajasthan have been fluctuating between –0.05 and 0.12. The active cases in this state have not increased too much in the latter part of April. An increase in recovery cases is one of the reasons. The estimated R_0_ for Rajasthan obtained from the fitted SIS model was 2.94. Therefore, the current COVID-19 situation in the state is not controlled yet.

#### Gujarat

**Figure 9:**
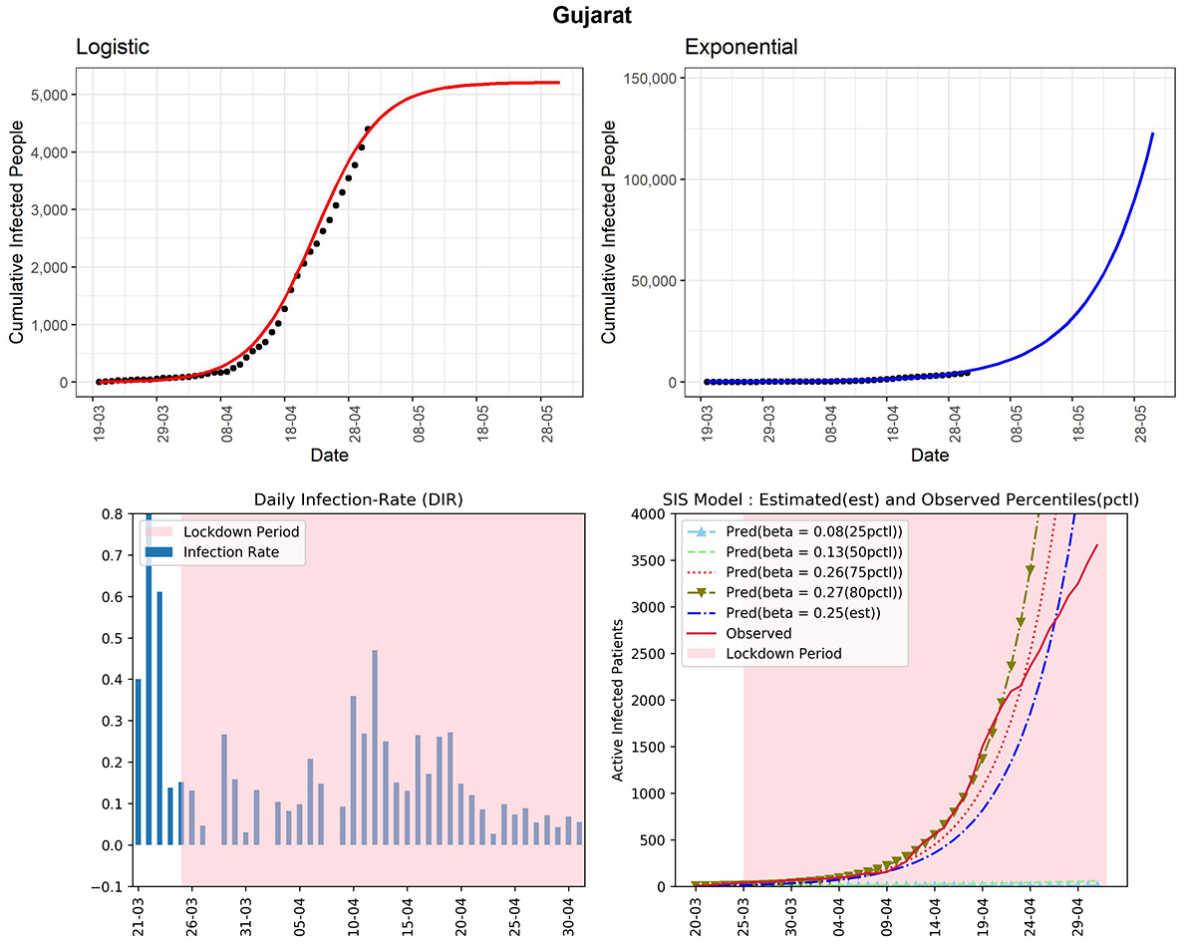
Graphs for the state of Gujarat. SIS: susceptible-infectious-susceptible.

The state is currently experiencing exponential growth with 4395 as the cumulative number of COVID-19 cases (see Figure 9). Using the logistic model, the cumulative infected cases could reach around 5206 in the next 30 days. There is apparently a stable rather than a declining trend in the DIRs in the last few days. The DIRs were in the range of 0.03-0.27 in the last 2 weeks, which are on the higher side. The curve (redline, fourth panel) of observed active infected patients is close to the curve of the SIS model corresponding to the estimated 75th percentile of observed DIR (β=.26). Surprisingly, in the latter part of the lockdown, the red line is experiencing exponential growth. The estimated R_0_ for Gujarat obtained from the fitted SIS model was 3.5, which is one of the highest. This state needs immediate intervention to implement all the preventive measures already taken by the Government strictly.

#### Uttar Pradesh

**Figure 10:**
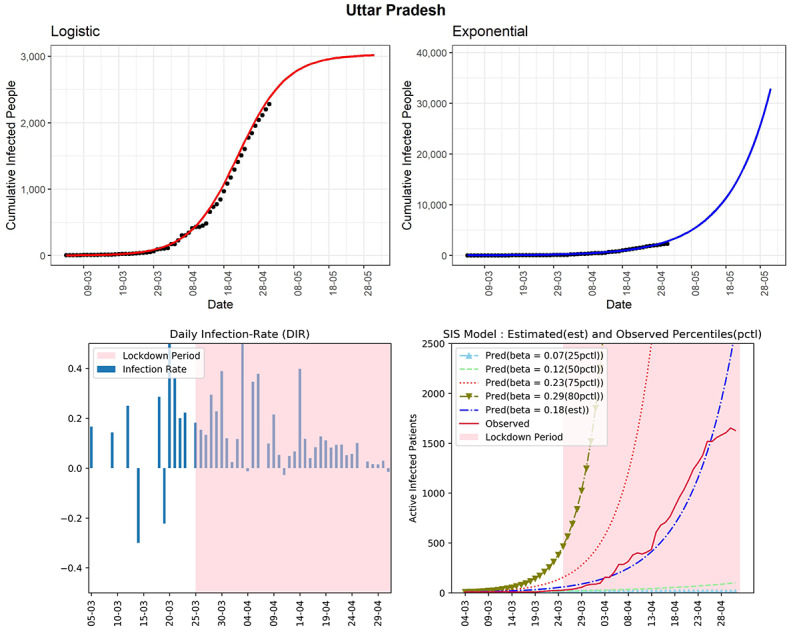
Graphs for the state of Uttar Pradesh. SIS: susceptible-infectious-susceptible.

This northern state of India has experienced 2281 cumulative COVID-19 cases (see Figure 10). Using the logistic model, the predicted number of cumulative confirmed cases could be around 3000 in the next 30 days. The curve (red line, fourth panel) of observed active infected patients was in between the curves of the SIS model corresponding to the 50th and 75th percentiles of observed DIRs (β=0.12 and 0.23, respectively). The DIR was in the range of –0.02 to 0.13 without a moderately decreasing trend in the last 2 weeks. The overall growth of active cases was still exponential, which is a major concern for the state. The estimated R_0_ for the state obtained from the fitted SIS model was 2.52. There could be many unreported cases in the state. In the absence of preventive measures, unreported cases can contribute to spreading the virus in the community.

#### Telangana

**Figure 11:**
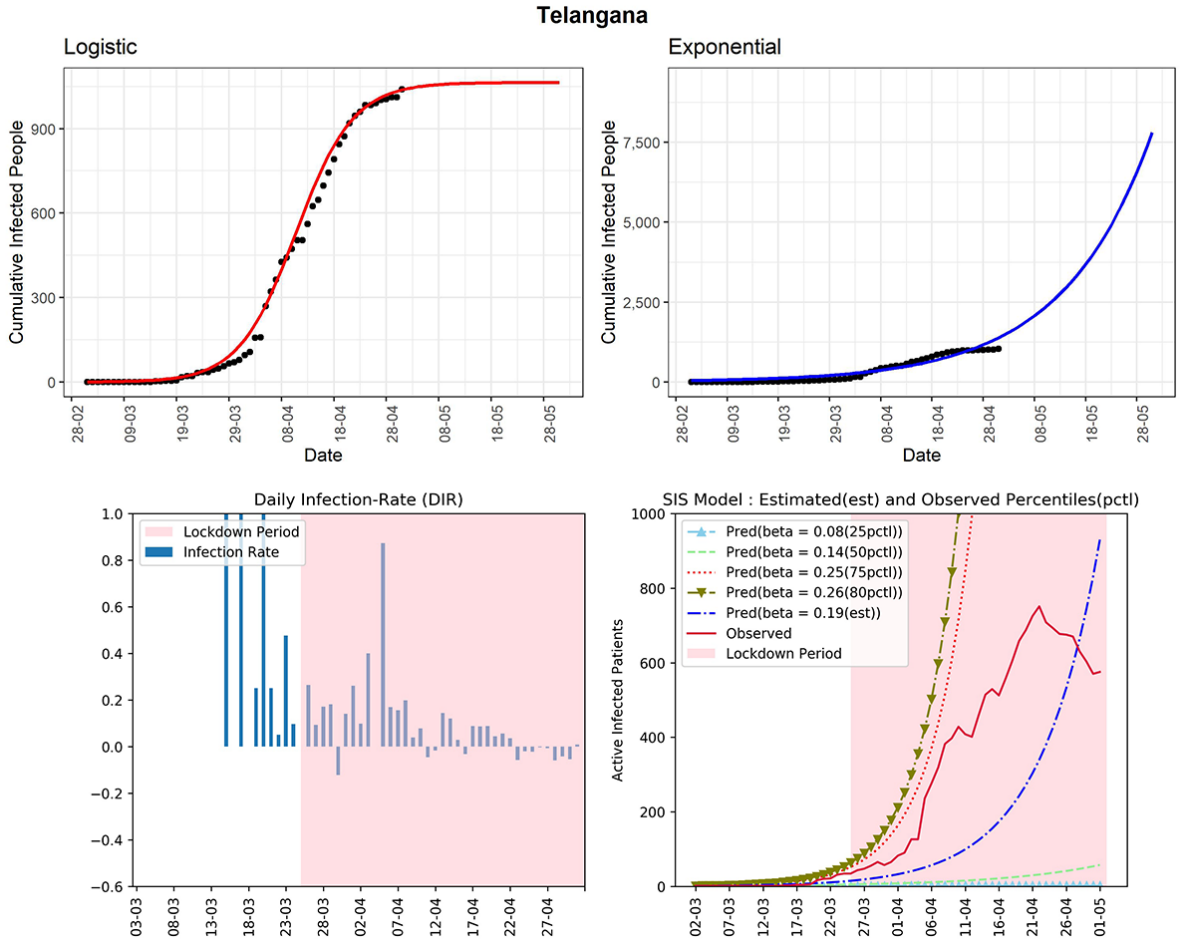
Graphs for the state of Telangana. SIS: susceptible-infectious-susceptible.

The southern Indian state of Telangana has reported 1039 cumulative infected cases until now (see Figure 11). The logistic model predicts that the number of cases for the state will be around 1063 in the next 30 days. In the fourth graph, the curve (red line, fourth panel) shows that the active number of cases has continuously remained below the curve of the SIS model corresponding to the 75th percentile of the observed DIRs (β=0.25). The estimated R_0_ for Telangana obtained from the fitted SIS model was 2.66. From April 23, 2020, onwards, there is a visible downward trend in the same line graph. This evidence is also supported by a clear decreasing trend in the DIR for more than 2 weeks. The state is going in the right direction to control the COVID-19 pandemic. However, preventive measures need to be in place to see long-term success against the virus.

#### Andhra Pradesh

**Figure 12:**
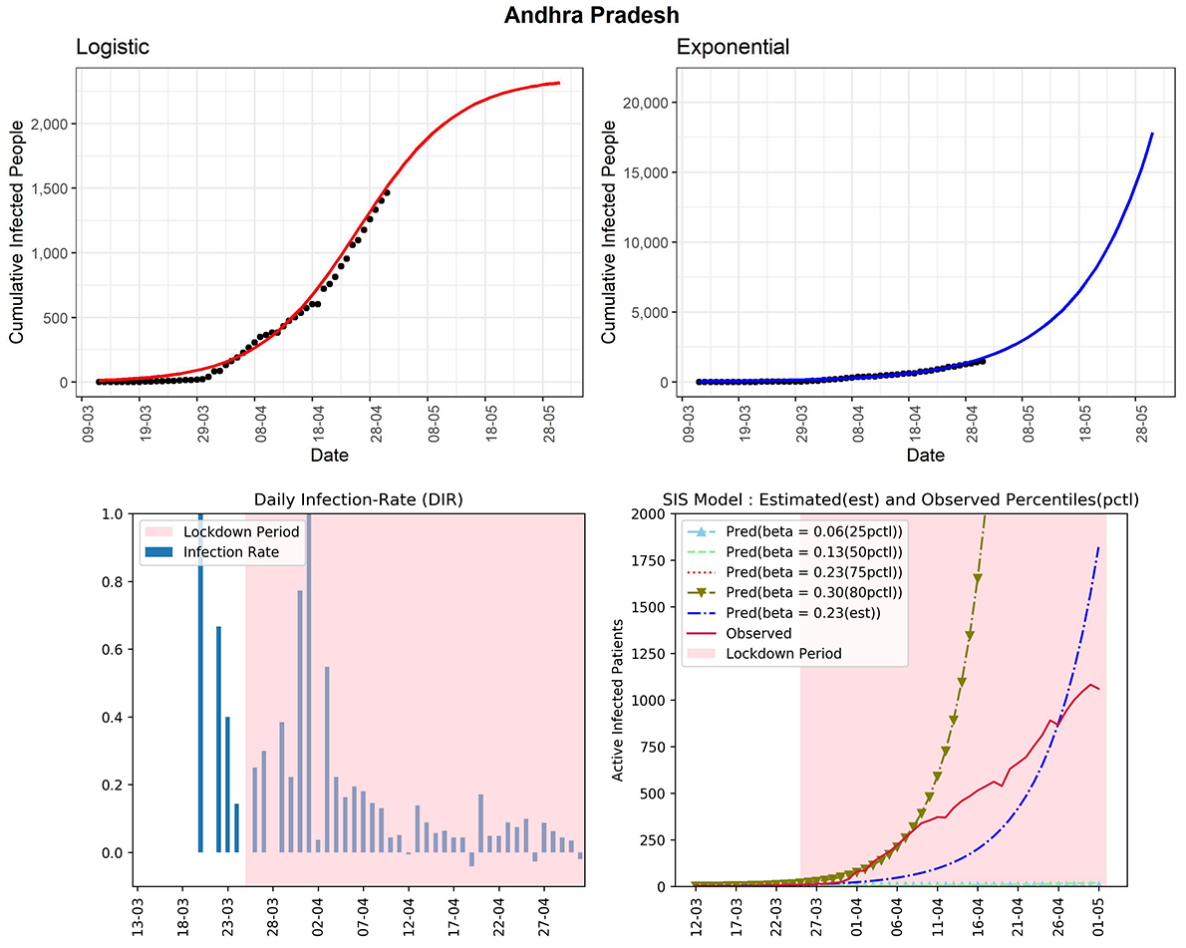
Graphs for the state of Andhra Pradesh. SIS: susceptible-infectious-susceptible.

This state has observed 1463 confirmed cumulative infected cases so far (see Figure 12). The curve (red line, fourth panel) shows that the number of active cases is now below and close to the curve of the SIS model corresponding to the 75th percentile of the observed DIR (β=0.23). The logistic model predicted that the maximum number of cumulative infected people will be around 2313 in the next 30 days. Despite showing good progress in mid-April, the state is again showing an exponential type growth rate. This state has seen DIRs between –0.04 and 0.17 during the last 2 weeks. The estimated R_0_ for this state obtained from the fitted SIS model was 3.22, which is quite high. The state has shown a few short declining trends, without any long-term declining trend in the DIR values. It could be due to many unreported infected cases in the community that is spreading the virus.

#### Kerala

**Figure 13:**
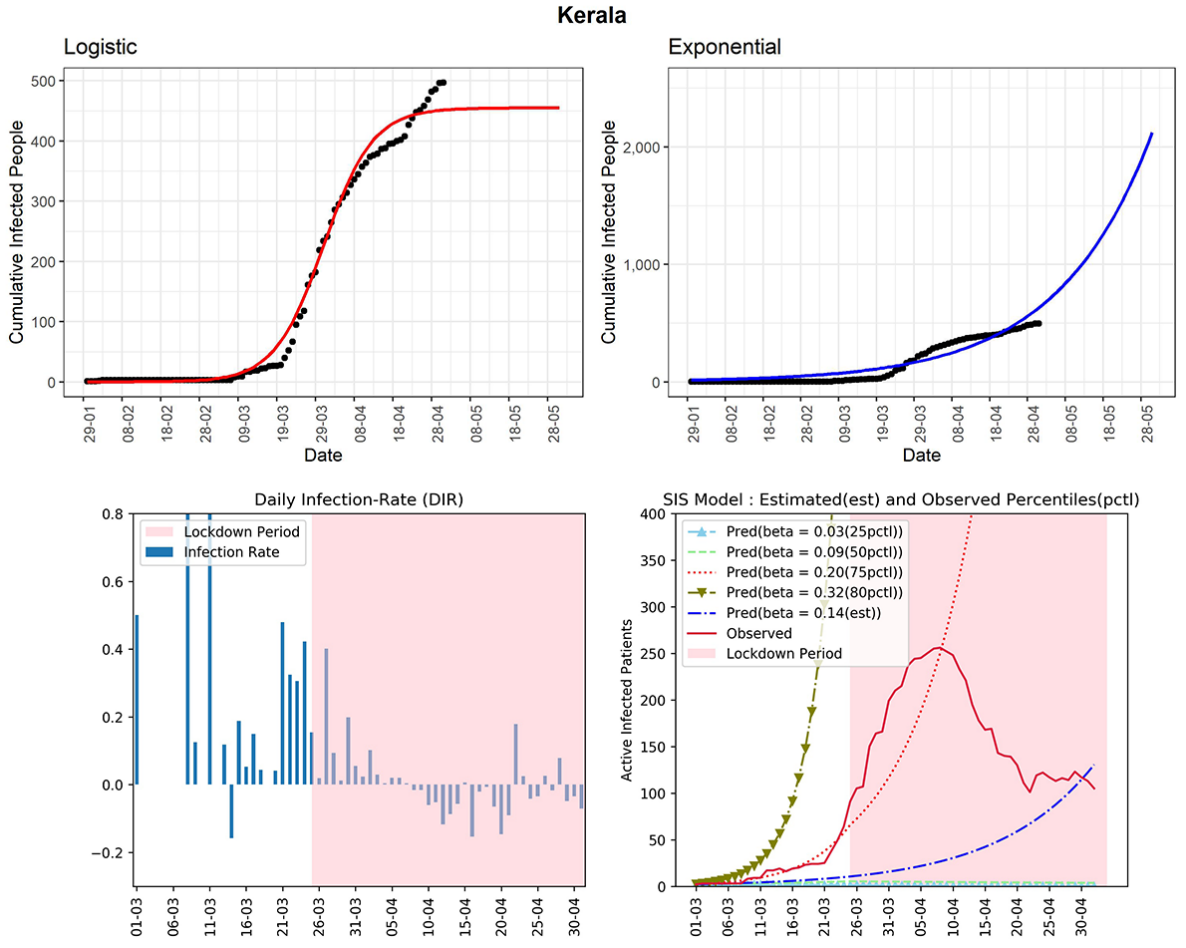
Graphs for the state of Kerala. SIS: susceptible-infectious-susceptible.

The southern state of Kerala is one of the few states of India where the effect of the lockdown is observed strongly. The state reported the first COVID-19 case in India. However, Kerala has been able to control the spread of the virus to a large extent to date. The cumulative number of cases reported until now is 497 (see Figure 13). It is a state where the curve (red line, fourth panel) of observed active infected patients is going down, which shows that the lockdown and other preventive measures have been effective for this state. The DIR has declined steadily from positive to negative values. However, some spikes in the DIR values can be noticed in the last few days. The estimated R_0_ for Kerala obtained from the fitted SIS model was 1.96, which is quite low compared to other states. It can be expected that with the present scenario of the extended lockdown the number of active cases will be few at the end of May.

#### Karnataka

**Figure 14:**
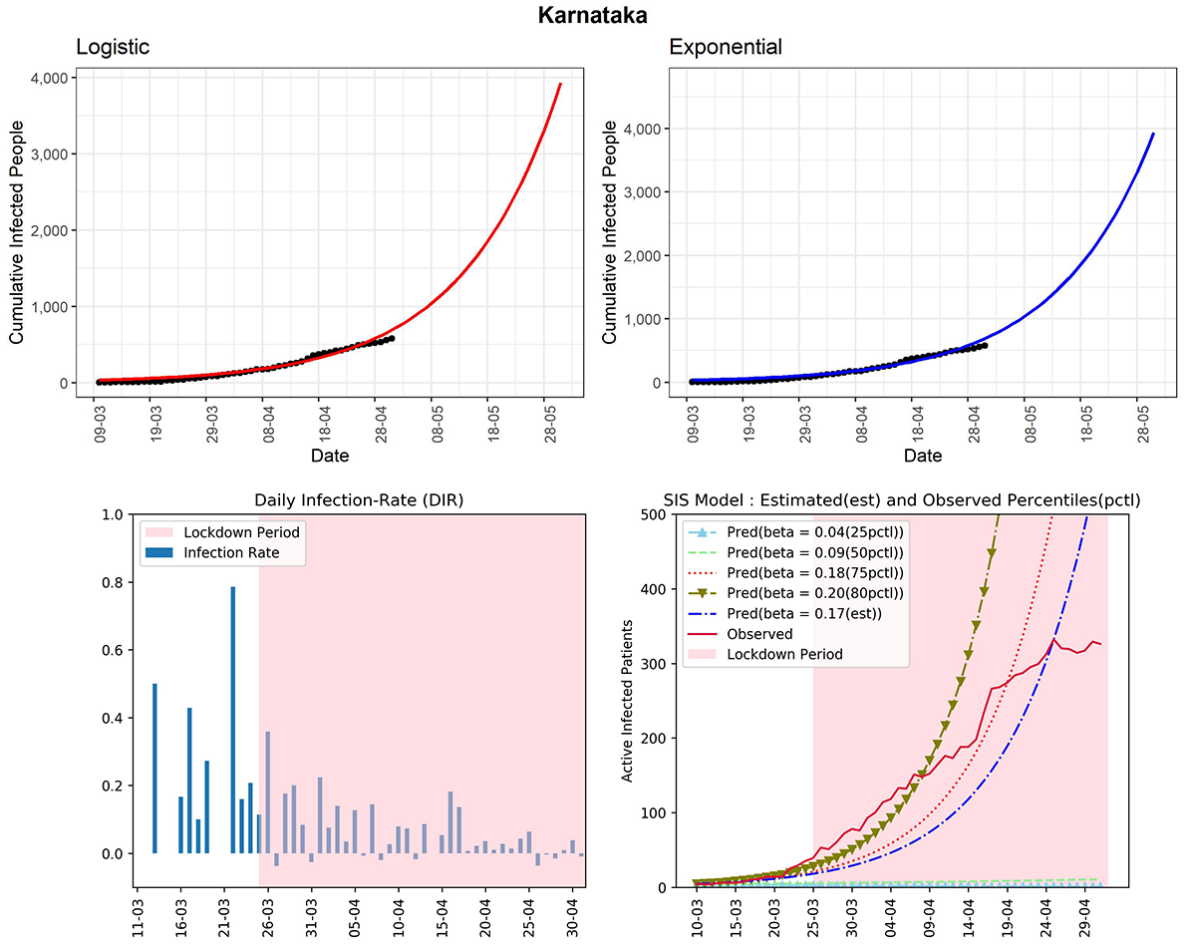
Graphs for the state of Karnataka. SIS: susceptible-infectious-susceptible.

The state has managed to restrict the cumulative infected cases to 576 until now (see Figure 14). The curve (red line, fourth panel) of observed active infected patients is now below the curve of the SIS model corresponding to the 75th percentile of the observed DIRs (β=0.18). Compared to other states, the 75th percentile DIR is on the lower side. The estimated R_0_ for the state obtained from the fitted SIS model was 2.38. We can observe the ups and downs of the DIR with an upper bound of 0.2 from early April. This state has seen DIRs between –0.04 and 0.06 during the last 2 weeks. However, the preventive measures need to be maintained to control the spread of the virus.

#### Jammu and Kashmir

**Figure 15:**
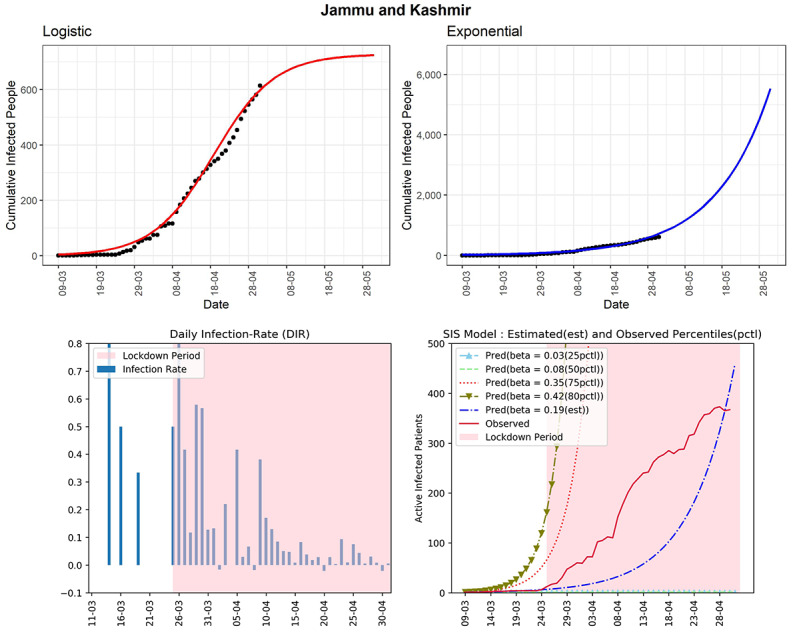
Graphs for the state of Jammu and Kashmir. SIS: susceptible-infectious-susceptible.

The northernmost state of Jammu and Kashmir has seen 614 cumulative infected cases so far (see Figure 15). The curve (red line, fourth panel) of observed active infected patients has been far below the curve of the SIS model corresponding to the 75th percentile of the observed DIR (β=0.35). The estimated R_0_ for the state obtained from the fitted SIS model was 2.66. From April 9, 2020, onwards, the DIR was apparently decreasing. There are some spikes in DIR values occasionally. It could be due to many unreported cases, which are allowing the infection to spread even during the lockdown period. The DIR was in the range of –0.02 to 0.09 in the last 2 weeks.

#### West Bengal

**Figure 16:**
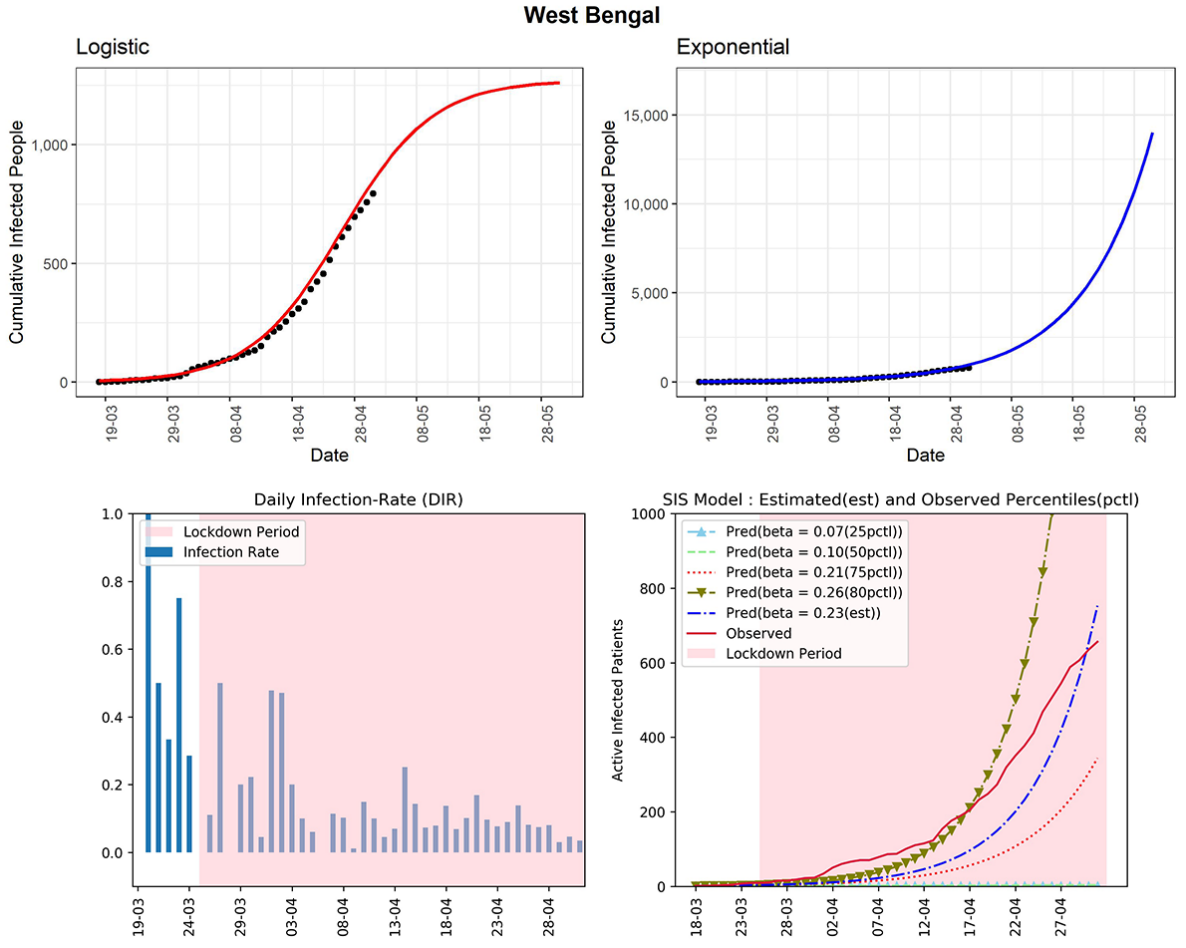
Graphs for the state of West Bengal. SIS: susceptible-infectious-susceptible.

The state of West Bengal is standing at 795 cumulative infected cases as of now (see Figure 16). The DIR values do not show any trend of slowing down in recent times. Based on the logistic model, the predicted cumulative infected cases could be around 1261 in the next 30 days. The curve (red line, fourth panel) of observed active infected patients was above the curve of the SIS model corresponding to the 75th percentile of the DIR (β=0.21). The DIRs were between 0.03 and 0.17 in the last 2 weeks. The cumulative infected cases graphs based on logistic and exponential models (first and second panels), as well as the active cases–based curve (red line, fourth panel) were all showing exponential type growth rates. The estimated R_0_ for West Bengal obtained from the fitted SIS model was 3.22, which is quite high. Strict implementation of preventive measures is needed to control the spread of COVID-19 in the state.

#### Haryana

**Figure 17:**
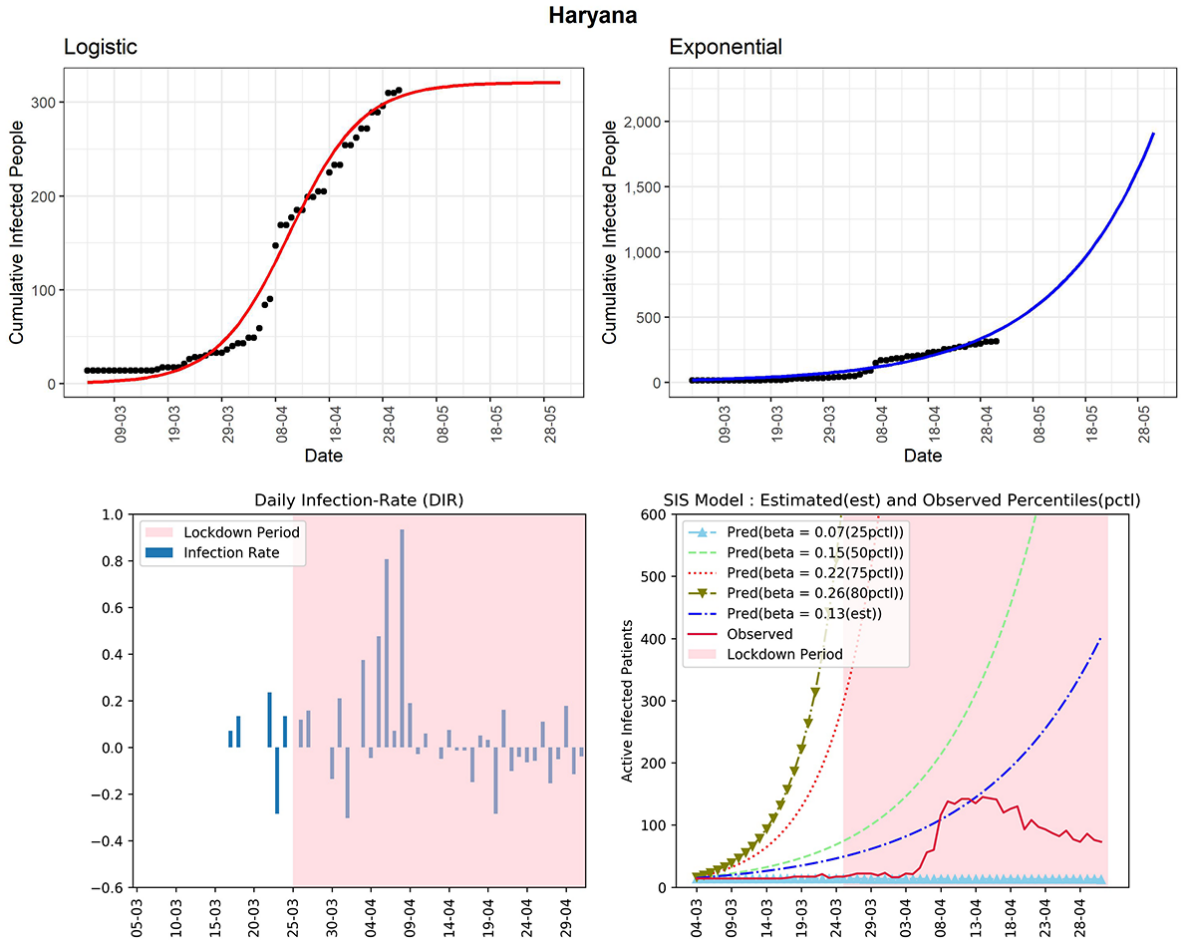
Graphs for the state of Haryana. SIS: susceptible-infectious-susceptible.

The state of Haryana has observed 313 cumulative infected COVID-19 cases so far (see Figure 17). It has reported a very low rate of infection in the latter part of the lockdown except for the last reported day. In the fourth panel, the curve (red line) of observed active infected patients is now far below the curve of the SIS model corresponding to the 50th percentile of observed DIRs (β=0.15) and is showing a decreasing trend in the latter part. The estimated R_0_ for the state obtained from the fitted SIS model was 1.82, which is on the lower side. The DIRs were between –0.28 and 0.18 in the last 2 weeks. Under the assumption that there are not too many unreported cases, the situation in Haryana seems to be under control.

#### Punjab

**Figure 18:**
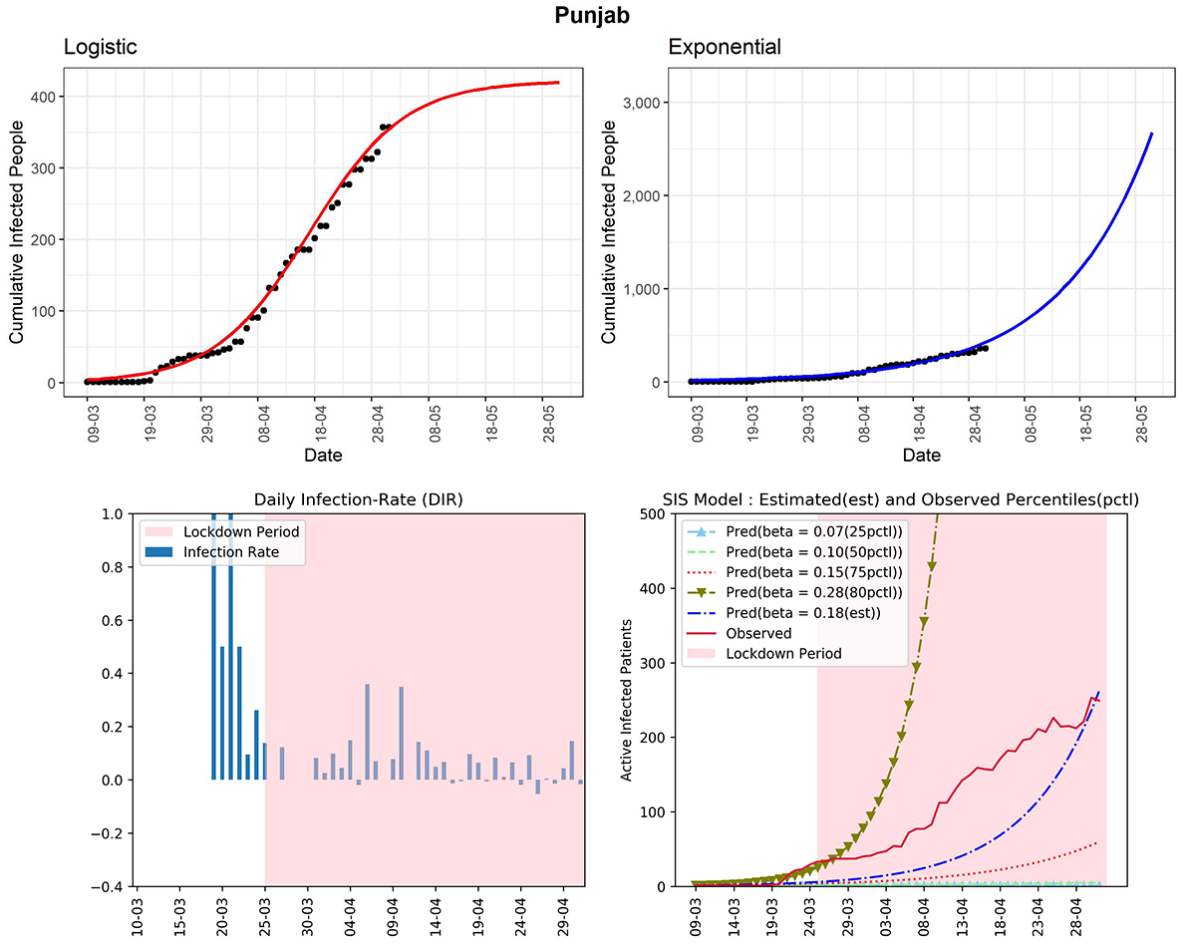
Graphs for the state of Punjab. SIS: susceptible-infectious-susceptible.

The state of Punjab has reported 357 cumulative infected cases until now (see Figure 18). Based on the logistic model, the predicted cumulative confirmed cases could be around 419 in the next 30 days. The curve (red line) of observed active infected patients was in between the SIS model curves corresponding to the estimated 75th and 80th percentiles of observed DIRs (β=0.15 and 0.28, respectively). The estimated R_0_ for Punjab obtained from the fitted SIS model was 2.52. The DIRs were between –0.05 and 0.14 in the last 2 weeks, which is good given the low number of active infected cases in the state.

#### Bihar

**Figure 19:**
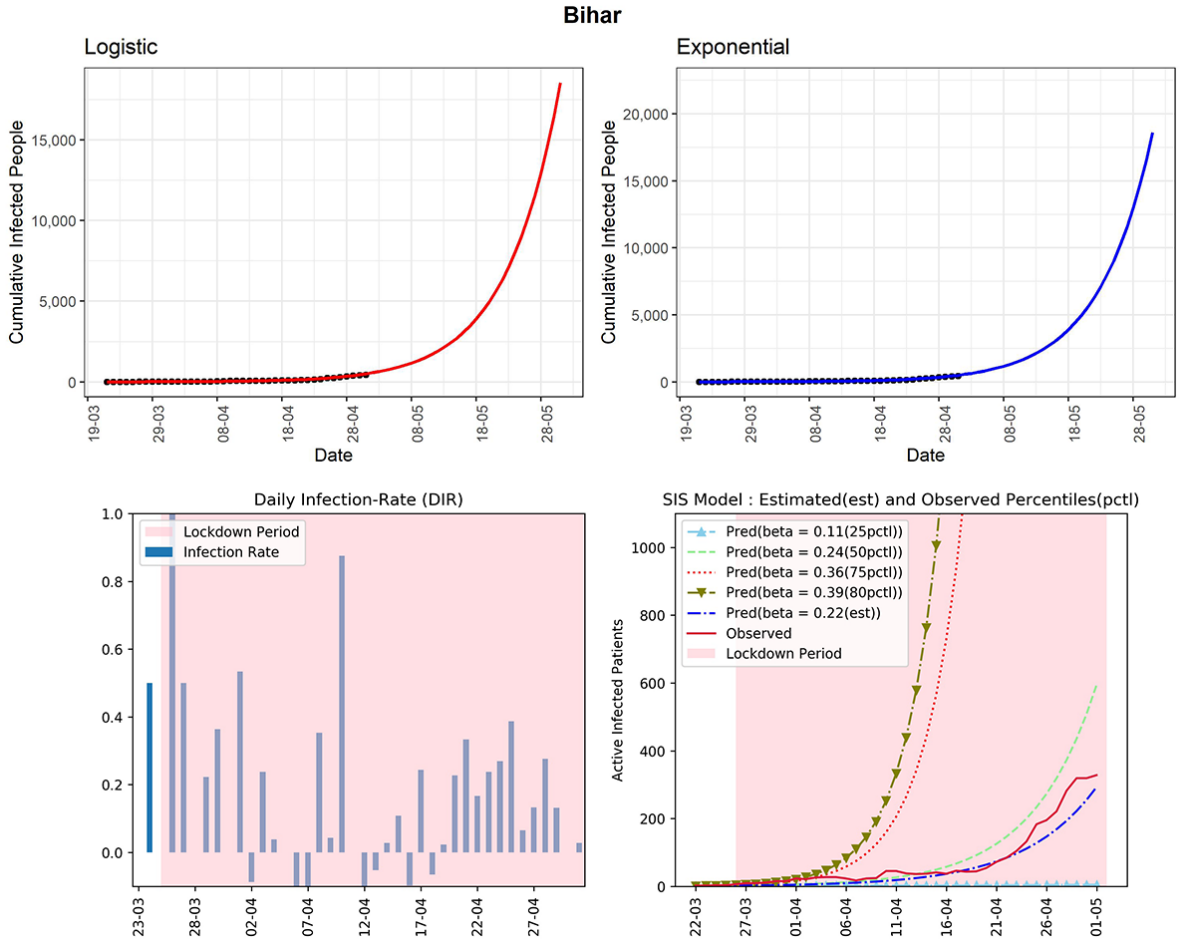
Graphs for the state of Bihar. SIS: susceptible-infectious-susceptible.

The state has reported 426 cumulative infected cases until now (see Figure 19). Based on the logistic model, Bihar could see 16,452 total infected cases in the next 30 days. The estimated R_0_ for the state obtained from the fitted SIS model was 3.08. It may be an overestimate. However, the DIRs showed no sign to decline in the last 2 weeks, with the highest reported value of 0.39. It may indicate many unreported cases in the state. However, the cumulative infected cases are still low for this state. Effective implementation of preventive measures is needed for the state.

### Joint Interpretation of Results From all Models

We consider a data-driven assessment of the COVID-19 situation based on the growth of active cases in recent times (red line, fourth panel in each state plot) along with the DIR values for each state (see Table 1). We labeled the condition of a state as *severe* if we observed a nondecreasing trend in DIR values over the last 2 weeks and a near exponential growth in active infected cases, as *moderate* if we observed an almost decreasing trend in DIR values over the last 2 weeks and neither increasing nor decreasing growth in active infected cases, and as *controlled* if we observed a decreasing trend in the last 2 weeks’ DIR values and a decreasing growth in active infected cases. It can be noticed that the logistic model is underpredicting the next 30-day prediction, whereas the exponential model is overpredicting the same. As we have argued earlier, despite nationwide lockdown, people are out of their homes for essential businesses, which can contribute to the spreading of the virus. The maximum value of DIR in the last 2 weeks can capture how severely COVID-19 is spreading in recent times. Note that, for example, a DIR value of 0.10 cannot be interpreted in the same way for two different states with, for example, 500 and 5000 active cases. For the first state, we see 500 x 0.10 = 50 new cases, and for the second state, we observe 5000 x 0.10 = 500 new cases. In an attempt to capture these various subtleties in a realistic prediction, we propose a linear combination prediction (LC_pred_) of the logistic and the exponential predictions using the maximum value of DIR over the last 2 weeks (DIR_max_) as a weighting coefficient (tuning parameter) as follows:

LC_pred_ = Logistic-prediction × (1 – λ) + Exponential-prediction × λ, where λ = max {0, min {1, DIR_max_}}

Such a choice of the tuning parameter λ makes the LC_pred_ equal to the logistic prediction when DIR_max_ is negative with λ=0. On the other hand, the LC_pred_ is equal to the exponential prediction when DIR_max_ is more than 1 with λ=1. When DIR_max_ is in between 0 and 1, the LC_pred_ is a combination of the predictions from the logistic and the exponential models. Given the situation in the entirety India, we recommend LC_pred_ along with the exponential predictions (particularly for states in severe condition) to be used for assessment purposes in each state.

Extensive testing may not be logistically feasible given India’s large population and limited health care budget. The undertesting can significantly impact the logistic prediction and less so the exponential prediction since the first one is underforecasting and the second one is overforecasting. The DIR indirectly captures the undertesting phenomenon. Thus, the LC_pred_ with (a truncated version of) DIR as the weight (λ) can be thought of as a treatment for undertesting, albeit in a limited fashion.

From Table 1, we can see that out of 16 states for which we have predictions, 10 states lay between the linear combination (LC_pred_) and the exponential predictions, 4 states are below the LC_preds_, and 2 states are above the exponential predictions.

## Discussion

India, a country of approximately 1.3 billion people, has reported 17,615 confirmed COVID-19 cases after 80 days (from January 30, 2020) from the first reported case in Kerala [36]. In a similar duration from the first case, the United States reported more than 400,000 cases, and both Spain and Italy reported more than 150,000 confirmed COVID-19 cases. To gain some more perspective, note that, the United States has around one-fourth of the Indian population size. Therefore, according to the reported data so far, India seems to have managed the COVID-19 pandemic better compared to many other countries. One can argue that India has conducted too few tests compared to its population size [37]. However, a smaller number of testing may not be the only reason behind the low number of COVID-19–confirmed cases in India so far. India has taken many preventive measures to combat COVID-19 in much earlier stages compared to other countries, including a nationwide lockdown from March 25, 2020. Apart from the lockdown, people have certain conjectures about possible reasons behind India’s relative success (eg, measures like the travel ban relatively early, use of Bacille Calmette-Guerin vaccination to combat tuberculosis in the population that may have secondary effects against COVID-19 [38,39], exposure to malaria and antimalarial drugs [40], and hot and humid weather slowing the transmission [41,42]). However, as of now, there is no concrete evidence to support these conjectures, although some clinical trials are currently underway to investigate some of these [43].

Note that India may have seen fewer COVID-19 cases until now, but the war is not over yet. There are many states like Maharashtra, Delhi, Madhya Pradesh, Rajasthan, Gujarat, Uttar Pradesh, and West Bengal who are still at high risk. These states may see a significant increase in confirmed COVID-19 cases in the coming days if preventive measures are not implemented properly. On the positive side, Kerala has shown how to effectively “flatten” or even “crush the curve” of COVID-19 cases. We hope India can limit the spread and impact of COVID-19 with a strong determination in policies as already shown by the central and state governments.

There are a few other works that are based explicitly on Indian COVID-19 data. Das [30] has used the epidemiological model to estimate the R_0_ at national and some state levels. Ray et al [44] used a predictive model for case counts in India. They also discussed hypothetical interventions with various intensities and provided projections over a time horizon. Both the papers have used the susceptible-infected-recovered model (or some extension) for their analysis and prediction. As we discussed earlier, considering the great diversity in every aspect of India, along with its vast population, it would be a better idea to look at each of the states individually. The study of each of the states individually would help decide further actions to contain the spread of the disease, which can be crucial for the specific states only. In this paper, we have mainly focused on the SIS model along with the logistic and the exponential models at each state (restricting to only those states with enough data for prediction). The SIS model takes into account the possibility that an infected individual can return to the susceptible class on recovery because the disease confers no long-standing immunity against reinfection. In South Korea, the health authorities discovered 163 patients who tested positive again after a full recovery [45,46]. The WHO is aware of these reports of patients who were first tested negative for COVID-19 using polymerase chain reaction testing and then after some days, tested positive again [47]. In a scientific brief, dated April 24, 2020, the WHO said, “there is currently no evidence that people who have recovered from COVID-19 and have antibodies are protected from a second infection” [48]. Several research papers have reported that, even though being infected by the virus may build immunity against the disease in the short-term, it is not a guaranteed fact, and it may not be long-lasting protection [49-51].

A report based on one particular model can mislead us. Here, we have considered the exponential, the logistic, and the SIS models along with the DIR. We have interpreted the results jointly from all models rather than individually. We expect the DIR to be zero or negative to conclude that COVID-19 is not spreading in a certain state. Even a small positive DIR such as 0.01 indicates that the virus is still spreading in the community and can potentially increase the DIR anytime. The states without a decreasing trend in DIR and near exponential growth in active infected cases are Maharashtra, Delhi, Gujarat, Madhya Pradesh, Andhra Pradesh, Uttar Pradesh, and West Bengal. The states with an almost decreasing trend in DIR and nonincreasing growth in active infected cases are Tamil Nadu, Rajasthan, Punjab, and Bihar. The states with a decreasing trend in DIR and decreasing growth in active infected cases in the last few days are Kerala, Haryana, Jammu and Kashmir, Karnataka, and Telangana. States with nondecreasing DIR need to do much more in terms of the preventive measures immediately to combat the COVID-19 pandemic. On the other hand, the states with decreasing DIR can maintain the same status to see the DIR become zero or negative for a consecutive 14 days to be able to declare the end of the pandemic.

Based on the modeling approaches presented in this paper, we have developed a web application [52] to see the Indian statewise forecast based on recent data that is updated regularly. The web application also offers a 30-day prediction of cumulative cases at the pan-India level by summing up the predicted cumulative cases of considered states.

## Appendix

Multimedia Appendix 1 Supplementary material.

## Abbreviations

COVID-19: coronavirus disease
DIR: daily infection rate
DIR_max_: maximum value of daily infection rate over the last 2 weeks
LC_pred_: linear combination prediction
R_0_: basic reproduction number
SIS: susceptible-infectious-susceptible
WHO: World Health Organization
