# Supplementary material for: COVID-19 in India: Statewise Analysis and Prediction
Source: JMIR Public Health Surveill. 2020 Aug 12;6(3):e20341. doi: 10.2196/20341 (PMC7431238; doi:10.2196/20341)
Supplement: Multimedia Appendix 1 [file publichealth_v6i3e20341_app1.docx]

**Statistical Models**

***Exponential Model:*** A pandemic can show exponential growth at the initial stage. For example, at the early stage, the 2014-15 Ebola epidemic in West Africa had shown a seemingly exponential spread. We can write the exponential model as

$y=y_{0}\times exp(\mu_{\max}\times time)$,

Where $y$ is the cumulative confirmed case at a specific time (date), $y_{0}$ is the initial population, $\mu_{max}$ is the maximum growth rate; time is the number of days from first confirmed infection.

***Logistic Model:*** Some pandemics follow an S-shaped curve (sigmoid curve). In other words, the pandemic may start slowly; then, it will increase the growth-rate (infection-rate), and finally, it will flatten the growth-rate over time. The following logistic model can capture that

$$y = \frac{K\times y_{0}}{y_{0}+\left( K-y_{0} \right)\times\exp\left( -\mu_{max}\times time \right)},$$

where $K$ is the maximum population size; other parameters have the same meaning as in the exponential model.

***Susceptible Infectious Susceptible (SIS) model:*** The SIS model is used for a given closed population that is susceptible to a particular disease, is prone to be infected, and communicate the infection within the community. It is a time dynamic model with the numbers of susceptible and infected people changing with time according to two different compartments which are characterized by two differential equations:

$\frac{dS}{dt}=-\frac{\beta SI}{N}+\gamma I$ ;

$$\frac{dI}{dt}=\frac{\beta SI}{N}-\gamma I.$$

In the above two differential equations, we are trying to observe the rate of change $\left( \frac{dS}{dt} \right)$ of the susceptible (S) population towards the inflection, and also the rate of change $\left( \frac{dI}{dt} \right)$ of the infected (I) persons. The model assumes two parameters, namely $\beta$, which is the average number of contacts per person per unit time, and $\gamma$, which is obtained as, $\gamma=\frac{1}{D}$, with $D$ being the recovery time (specifically, it is the time during which a particular patient can infect others). Here N denotes the total population size with N = S + I.

Table 3 Goodness of fit measures for the logistic and exponential models

| State | Logistic | | Exponential | |
| --- | --- | --- | --- | --- |
|  | R-square | Deviance | R-square | Deviance |
| Andhra Pradesh | 0.99 | 70932.20 | 0.99 | 169609.12 |
| Bihar | 0.99 | 8244.24 | 0.99 | 8243.97 |
| Delhi | 0.99 | 563263.44 | 0.97 | 1979522.61 |
| Gujarat | 1.00 | 222500.23 | 0.98 | 1869895.38 |
| Haryana | 0.99 | 10970.46 | 0.93 | 49223.34 |
| Jammu and Kashmir | 0.99 | 14326.21 | 0.97 | 62115.67 |
| Karnataka | 0.97 | 61993.09 | 0.97 | 62001.89 |
| Kerala | 0.99 | 22603.01 | 0.91 | 287919.78 |
| Madhya Pradesh | 0.99 | 330231.58 | 0.96 | 1406825.35 |
| Maharashtra | 1.00 | 437647.58 | 0.99 | 4452503.81 |
| Punjab | 0.99 | 4082.03 | 0.97 | 24317.88 |
| Rajasthan | 1.00 | 58694.90 | 0.97 | 1379677.74 |
| Tamil Nadu | 0.99 | 357650.72 | 0.95 | 1740108.65 |
| Telangana | 1.00 | 29424.88 | 0.92 | 797659.71 |
| Uttar Pradesh | 1.00 | 33738.33 | 0.98 | 541768.49 |
| West Bengal | 1.00 | 4001.97 | 0.99 | 32894.14 |
